# Allele-specific effects of mutations in the rifampin resistance-determining region (RRDR) of RpoB on physiology and antibiotic resistance in *Enterococcus faecium*

**DOI:** 10.1128/msphere.00506-25

**Published:** 2025-12-04

**Authors:** Adeline Supandy, Emma G. Mills, Kyong T. Fam, Ryan K. Shields, Howard C. Hang, Daria Van Tyne

**Affiliations:** 1 Division of Infectious Diseases, University of Pittsburgh, School of Medicine 12317 , Pittsburgh, Pennsylvania, USA; 2 Department of Immunology and Microbiology, Scripps Research 478079 , La Jolla, California, USA; 3 Antibiotic Management Program, University of Pittsburgh Medical Center 6595, Pittsburgh, Pennsylvania, USA; 4 Department of Chemistry, Scripps Research 683716, La Jolla, California, USA; 5 Center for Evolutionary Biology and Medicine, University of Pittsburgh 6614, Pittsburgh, Pennsylvania, USA; University of Nebraska Medical Center, College of Medicine, Omaha, Nebraska, USA

**Keywords:** *Enterococcus faecium*, DNA-directed RNA polymerase, RpoB, functional genomics, transcriptomics, antibiotic resistance

## Abstract

*Enterococcus faecium* is a member of the human gut microbiota that has evolved into a problematic nosocomial pathogen and a leading cause of infections in hospitalized patients. Treatment of *E. faecium* infections is complicated by antibiotic resistance, making it important to understand resistance mechanisms and their broader consequences in this pathogen. Here, we explored the collateral effects of rifampin resistance-associated mutations in the *E. faecium* RNA polymerase β-subunit (RpoB). Of 14,384 publicly available *E. faecium* genomes, nearly one-third carried a mutation in the rifampin resistance-determining region (RRDR) of RpoB. In a local population of 710 *E. faecium* clinical isolates collected from patients at a single hospital, we found significant associations between the presence of RRDR mutations and prior exposure to rifamycin antibiotics, as well as associations between RRDR mutations and altered daptomycin susceptibility. To investigate the phenotypic impacts of RRDR mutations, we generated and studied four isogenic strains with distinct RRDR mutations (Q473K, G482D, H486Y, and S491L) that overlapped with clinical isolate variants. Transcriptomic and phenotypic analyses revealed allele-specific effects on *E. faecium* gene expression, growth dynamics, antibiotic susceptibility, isopropanol tolerance, and cell wall physiology. One frequently observed mutation, H486Y, caused minimal transcriptional changes and enhanced bacterial fitness under antibiotic stress. In contrast, the S491L mutation induced extensive transcriptional changes and slowed bacterial growth but also conferred increased isopropanol tolerance, potentially enhancing bacterial survival on hospital surfaces. Overall, our findings highlight the multifaceted impacts of RRDR mutations in shaping *E. faecium* physiology and antibiotic resistance, two important features of this hospital-associated pathogen.

**IMPORTANCE:**

Understanding how antimicrobial resistance affects bacterial physiology is critical for developing effective therapeutics against bacterial infections. In this study, we found that rifampin resistance-associated mutations in RpoB are widespread in *Enterococcus faecium,* a leading multidrug-resistant pathogen. By studying isogenic wild-type and RpoB mutant strains, we discovered that RpoB mutations, although conferring resistance to rifampin, have distinct allele-specific effects on other bacterial phenotypes. Some of these collateral effects appear to promote *E. faecium* resistance to antibiotics and survival in the hospital environment, raising questions about the selective pressures driving their emergence. Overall, our study underscores the importance of examining the collateral effects of resistance-associated mutations in multidrug-resistant pathogens, which could help mitigate their persistence and spread among vulnerable patients.

## INTRODUCTION

Antimicrobial resistance represents one of the most pressing public health challenges in the 21st century, with nearly 5 million deaths attributed to resistant infections each year (1, 2). Among the pathogens driving this crisis, *Enterococcus faecium* causes invasive infections that are often antibiotic-resistant and difficult to treat. The ability to rapidly adapt to diverse environments allows *E. faecium* to overcome bottlenecks caused by selective pressures such as antibiotic exposure, nutrient limitation, and host immunity. As such, among the ESKAPE pathogens, *E. faecium* is a leading cause of bloodstream infections (BSIs), especially in hospitalized and immunocompromised patients (3, 4). In addition to various intrinsic resistance mechanisms, *E. faecium* can rapidly acquire additional antibiotic resistance genes (5–7). Treatment is particularly challenging due to its ability to resist several important Gram-positive-targeting antibiotics, including vancomycin, ampicillin, and daptomycin (6–11). Consequently, *E. faecium* is often associated with treatment failure and recurrent infections, contributing to an estimated 100,000 deaths globally each year (12). Understanding the mechanisms underlying this adaptability is crucial to developing new therapeutics against *E. faecium* infections.

Although the genetic determinants of antibiotic resistance in *E. faecium* have been studied extensively, many resistance mechanisms have yet to be fully elucidated (5). In particular, recent studies have demonstrated that mutations in the DNA-dependent RNA polymerase β-subunit (RpoB), particularly in the 81 bp rifampin resistance-determining region (RRDR), may play a broader role beyond rifampin resistance (13, 14). For example, RRDR mutations have been associated with increased resistance to cephalosporins and daptomycin, the latter of which is a last-resort antibiotic for *E. faecium* infections (13, 14). These findings suggest that RRDR mutations may have pleiotropic effects on antibiotic susceptibility and resistance in *E. faecium*. Additionally, it is unclear whether the emergence of RRDR mutations in *E. faecium* is driven solely by exposure to rifamycins or whether other environmental factors might contribute to their selection. Because RpoB is an essential and highly conserved component of the bacterial transcriptional machinery, mutations in this protein are likely to affect a variety of cellular processes through altered gene expression (15). A deeper understanding of how RRDR mutations emerge and their impact on *E. faecium* physiology is crucial to the development of new treatments for *E. faecium* infections.

In this study, we investigated the prevalence, diversity, and functional impact of mutations in the RRDR of RpoB in *E. faecium*. We determined the global distribution of RRDR mutations and confirmed previously described associations between RRDR mutations and daptomycin susceptibility in a cohort of over 700 patients from a single hospital. We also used genomic, transcriptomic, and phenotypic analyses to investigate allele-specific effects of RRDR mutations on *E. faecium* physiology and resistance to antibiotics beyond rifampin. Taken together, our findings highlight the multifaceted roles of RRDR mutations in shaping *E. faecium* biology.

## RESULTS

### RRDR mutations are prevalent in human-associated *E. faecium*

**Fig 1:**
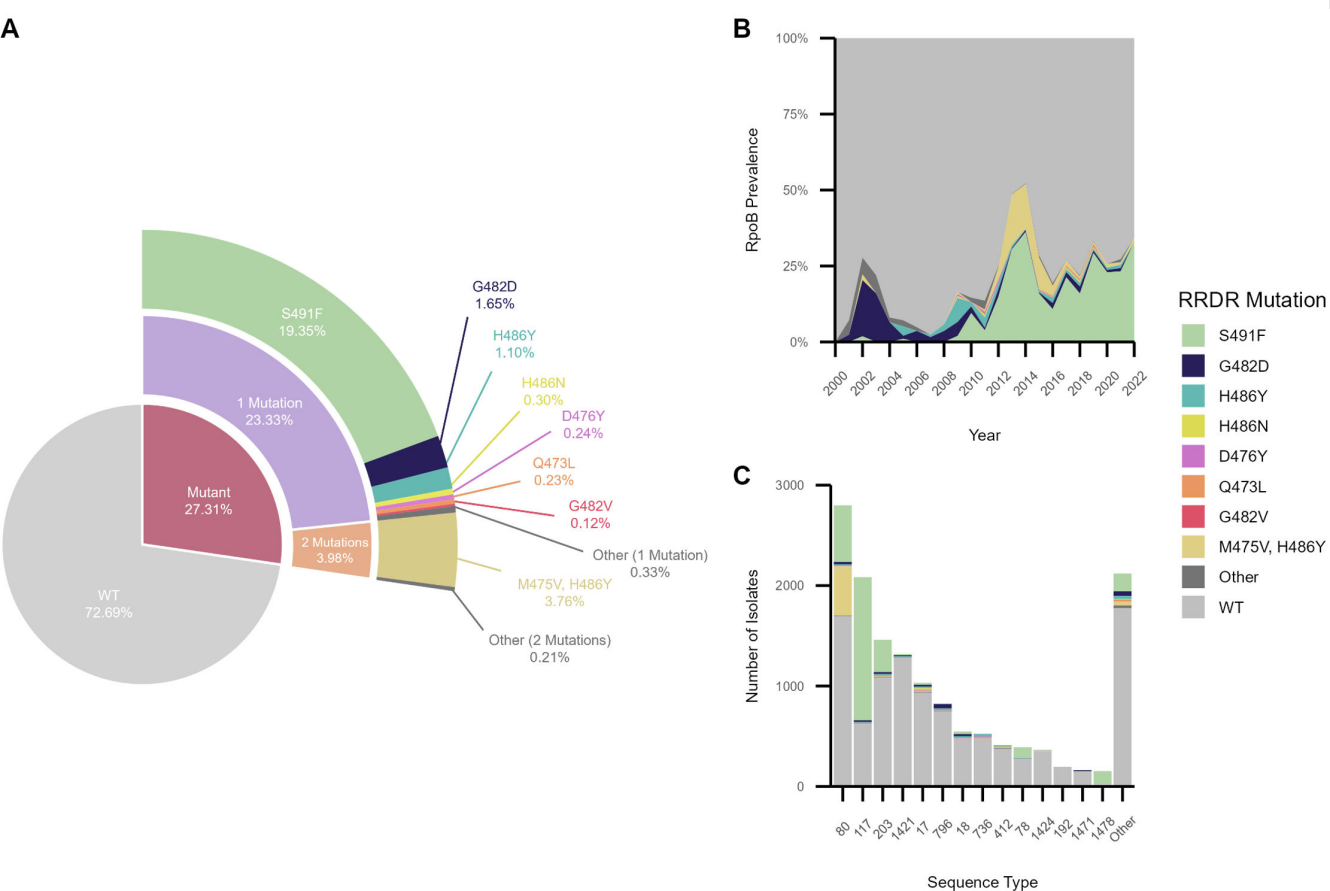
Distribution of mutations in the RpoB rifampin resistance-determining region (RRDR) across 14,384 publicly available *E. faecium* isolate genomes. RRDR variants were categorized based on total variant frequency (**A**), year of isolation (**B**), and sequence type (ST) (**C**). Data were collected from the NCBI and filtered according to Fig. S1 to include only human-associated *E. faecium* collected between 2000 and 2023.

To understand the global epidemiology of RRDR mutations in *E. faecium*, we analyzed all publicly available genomes deposited in the National Center for Biotechnology Information (NCBI) database between 2000 and 2023. The data set was filtered to include 14,384 human-associated *E. faecium* genomes, with most isolates originating from Europe (52%), North America (25%), and Oceania (16%) (Fig. S1 and S2, File S1). Approximately 30% of genomes encoded one or more mutations in the RRDR (Fig. 1A; Fig. S3). The most frequent mutation, S491F, was found in nearly 20% of all global *E. faecium* genomes and appeared to increase in frequency starting in 2007 (Fig. 1B). This and the second most prevalent variant, the H486Y/M475V double mutant, were frequently found among isolates belonging to the emerging lineages sequence type (ST) 80 and ST117 (Fig. 1C) (16). Many RRDR mutations were found across multiple countries, STs, and genetic backgrounds, suggesting independent emergence.

**Fig 2:**
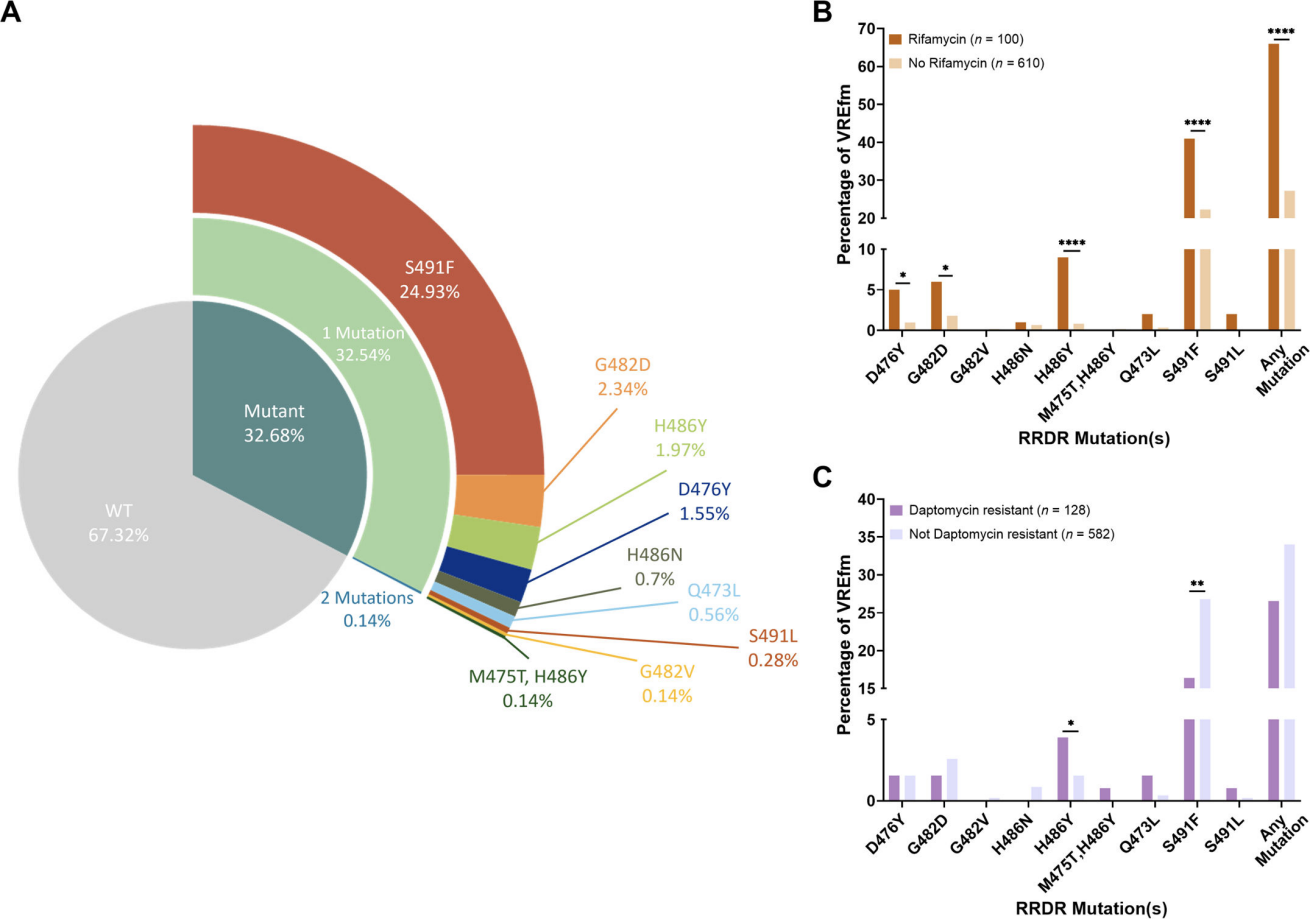
Associations between RRDR mutations, prior antibiotic exposure, and resistance in 710 vancomycin-resistant *E. faecium* (VREfm) isolates collected from a single medical center. (**A**) Distribution of RRDR mutations across VREfm isolates based on total variant frequency. (**B**) Associations between the percentage of VREfm isolates with RRDR mutations in patients with prior exposure to antibiotics in the rifamycin class (*n* = 100) or patients without exposure (*n* = 610). (**C**) Associations between the percentage of VREfm isolates with RRDR mutations that are daptomycin-resistant (*n* = 128) or daptomycin-susceptible (*n* = 582). Data were analyzed with one-way ANOVA. *P ≤ 0.05; **P ≤ 0.01; ****P ≤ 0.0001.

As RRDR mutations have been shown to confer rifampin resistance, we sought to assess the association between RRDR mutations and prior exposure to rifamycin class antibiotics among 710 *E. faecium* isolates collected from patients at the University of Pittsburgh Medical Center between 2017 and 2022 (16–19). Similar to the global distribution, roughly 33% of these isolates encoded RRDR mutations, with S491F being the most prevalent (25%) (Fig. 1A and 2A). Most of the RRDR mutations detected were strongly associated with prior exposure to rifamycin class antibiotics, including the D476Y, G482D, H486Y, and S491F variants (Fig. 2B). A prior study found that RRDR mutations were also associated with daptomycin resistance in *E. faecium* (14), and we wondered whether similar associations were present in this data set. Although prior exposure to daptomycin was not associated with RRDR mutations, we observed that two mutations were significantly associated with altered daptomycin susceptibility (Fig. 2C). The H486Y mutation was associated with increased daptomycin resistance; however, the S491F mutation was associated with daptomycin sensitivity. Our results are similar to the prior study (14) but reveal additional strain- and allele-specific effects of RRDR mutations on *E. faecium* fitness and antibiotic sensitivity.

### RRDR mutations induce widespread transcriptional changes in *E. faecium*

Since the RNA polymerase plays a critical role in gene transcription, we wondered how different RRDR mutations altered *E. faecium* transcriptional profiles at a genome-wide scale (20–23). To investigate this question, we generated four isogenic RRDR mutants through one-step selection by plating a clinical *E. faecium* isolate onto media containing rifampin (24). Whole-genome sequencing of individual clones confirmed that the resulting rifampin-resistant isolates were isogenic and carried G482D, Q473K, H486Y, or S491L mutations in the RRDR. The G482D and H486Y mutations were observed in the human-associated *E. faecium* data sets, whereas mutations at residues Q473 and S491 were also detected, albeit with substitutions to different amino acids (Fig. 1A and 2A).

**Fig 3:**
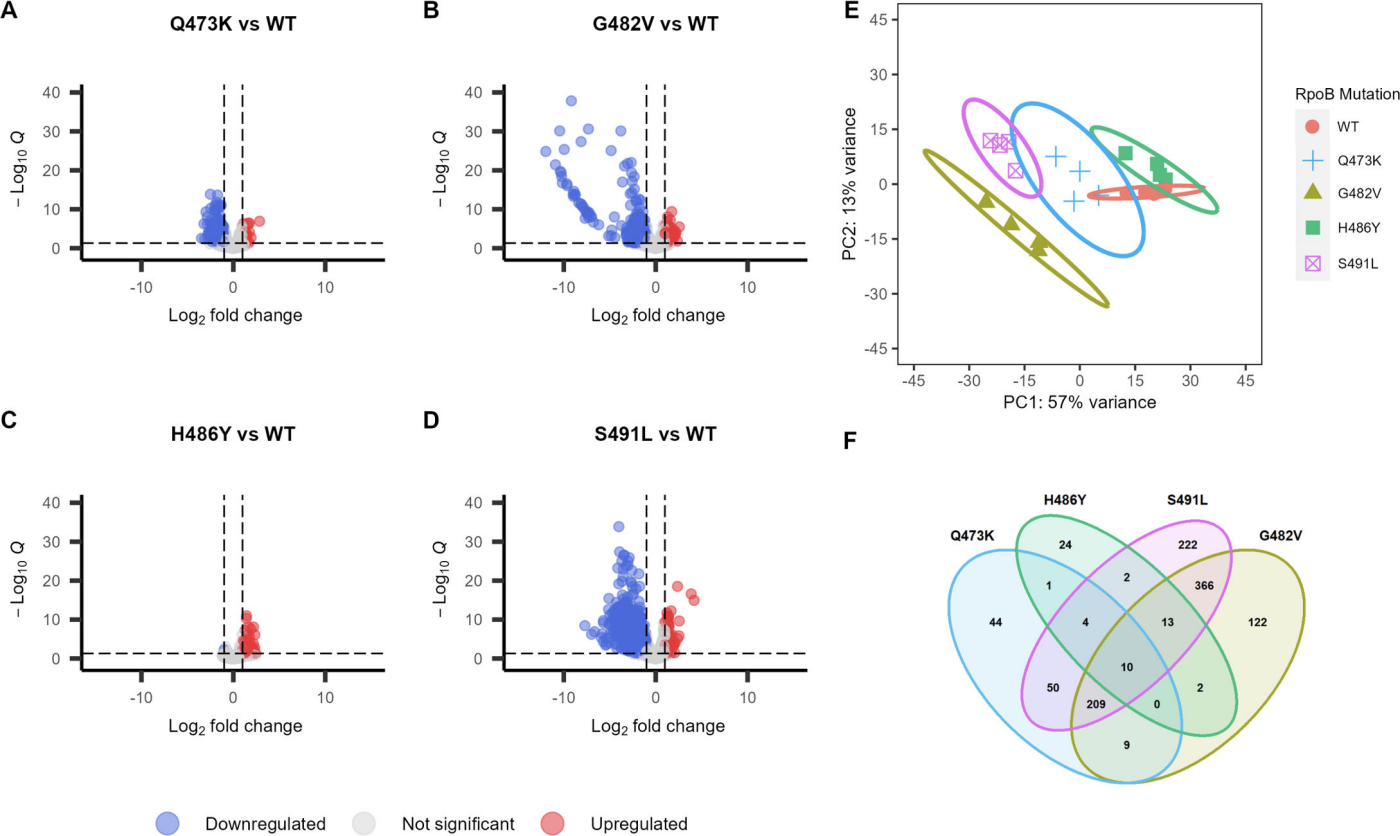
Visualization of differentially expressed genes (DEGs) due to mutations in RRDR as identified by RNA sequencing. (**A–D**) Volcano plots showing the distribution of up- and down-regulated genes in each RRDR mutant. The *x*-axis shows log_2_ fold change in expression level, and the *y*-axis shows the significance of the difference in expression. (**E**) Principal component analysis of gene expression data with 95% confidence intervals circled. Different RRDR mutants are represented by different colors and shapes. Each dot represents one biological replicate. (**F**) Venn diagram showing the number of shared differentially expressed genes between RRDR mutant strains.

To measure the effects of different RRDR mutations on *E. faecium* transcription*,* we performed RNA sequencing on the wild-type (WT) and isogenic mutant strains and identified differentially expressed genes in each mutant (Fig. 3A through D). The overall number of differentially expressed genes in each mutant strain varied widely, with many genes downregulated in the Q473K, G482V, and S491L mutants and a small number of genes upregulated in the H486Y mutant. Principal component analysis revealed tight clustering of replicate transcriptomes of each strain; however, transcriptomes of different strains clustered apart from one another (Fig. 3E). H486Y mutant transcriptomes clustered closely with those of the WT strain, consistent with the lower number of differentially expressed genes in that strain (Fig. 3E and F). We also compared the differentially expressed genes in the mutant strains to one another and found that many genes were unique to each mutant (Fig. 3F; Fig. S4A and B). Ten genes were differentially expressed in all four mutant strains (Fig. 3F); however, these genes were all downregulated in the Q473K, G482V, and S491L mutants but upregulated in the H486Y mutant (File S1).

**Fig 4:**
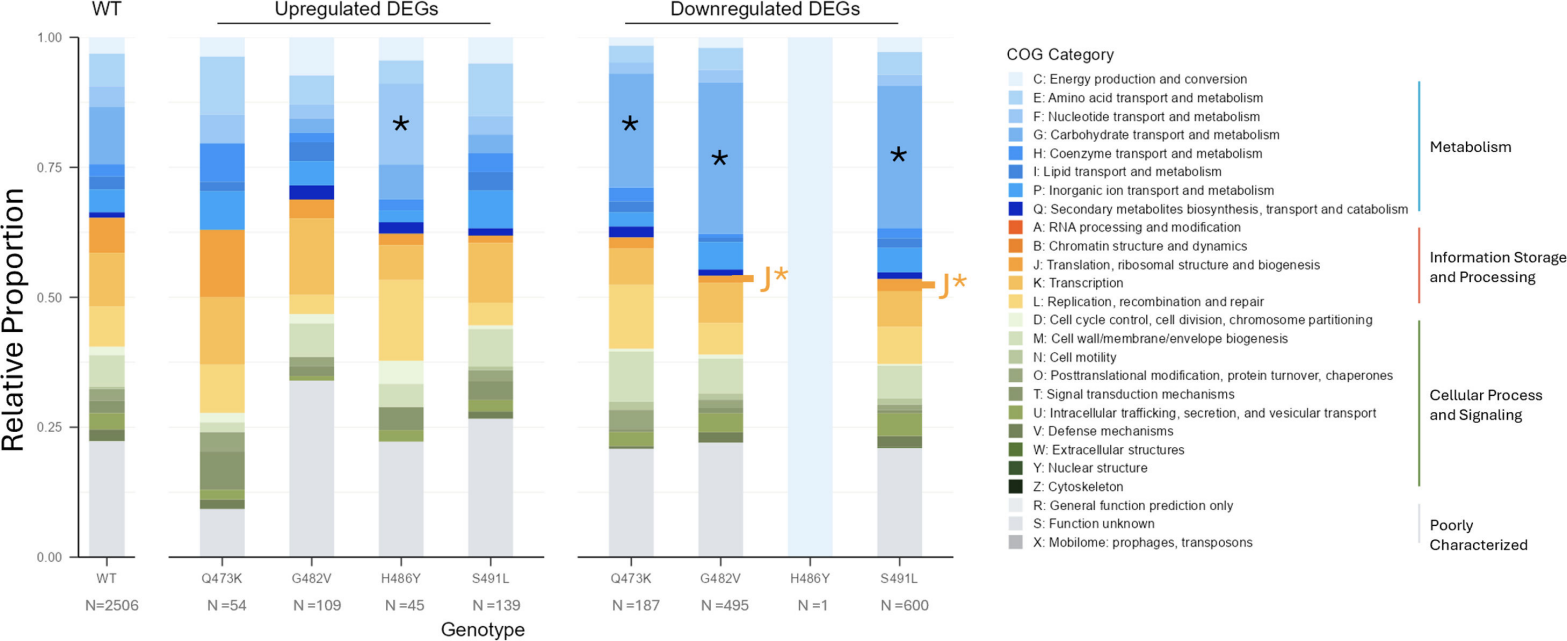
Distribution of COG categories in the wild-type (WT) strain genome and among differentially expressed genes in each RRDR mutant strain. COG categories were assigned using EggNOG-mapper; genes that did not have assigned COG categories were excluded. The distribution of differentially expressed genes in each RRDR mutant strain was compared with the distribution of all genes in the WT strain genome using Fisher’s exact test with Bonferroni correction. *P ≤ 0.05.

We next investigated the possible functional roles of differentially expressed genes in each mutant strain by analyzing their distribution of Clusters of Orthologous Groups (COG) categories relative to the distribution of all genes in the WT strain genome (25, 26). Among upregulated genes, only COG category F (Nucleotide Transport and Metabolism) was significantly enriched in the H486Y mutant (Fig. 4). The H486Y mutant had a single gene downregulated, whereas the other three mutant strains all showed significant downregulation of genes involved in Carbohydrate Transport and Metabolism (Category G; Fig. 4). Both the S491L and G482V mutants also demonstrated significant down-regulation of genes related to Translation, Ribosome Structure, and Biogenesis (Category J; Fig. 4). Together, these findings suggest that RRDR mutations differentially affect global transcription and primarily target metabolic pathways, potentially impacting *E. faecium* fitness and growth dynamics.

### Allele-specific effects of RRDR mutations on *E. faecium* growth rate and antimicrobial susceptibility

**Fig 5:**
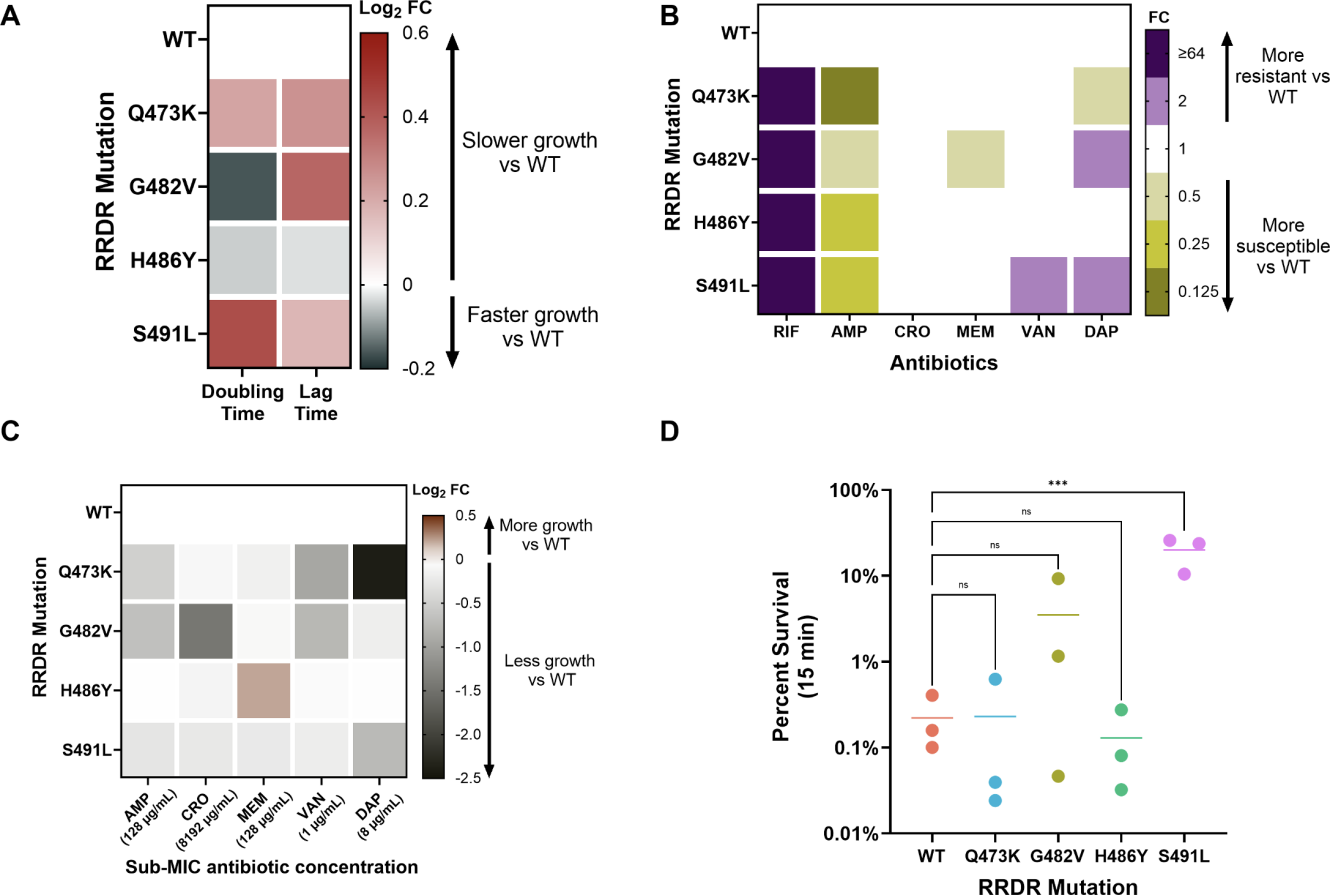
Allele-specific effects of RRDR mutations on *E. faecium* phenotypes. (**A**) Doubling and lag time of each strain were determined through growth assays in rich media. Results are shown as a heatmap of log_2_ fold-change (FC) compared to the wild-type (WT) strain. (**B**) MICs determined by broth microdilution. The MIC of each RRDR mutant is shown as FC compared with WT. (**C**) RRDR mutant growth under antibiotic stress, shown as the log_2_ FC of the total area under the growth curve (AUC) compared with WT. (**D**) Isopropanol tolerance is displayed as percent survival after 15 min incubation with 20% isopropanol. All experiments were conducted in triplicate, and mean values are shown. Significance was calculated with one-way ANOVA. ***P ≤ 0.001; ns, not significant. RIF, rifampin; AMP, ampicillin; CRO, ceftriaxone; MEM, meropenem; VAN, vancomycin; DAP, daptomycin.

Although RRDR mutations primarily decrease rifampin binding (18, 27), these mutations can also impact RNA synthesis and thus overall bacterial growth rates (20, 28–31). We assessed the impact of RRDR-mediated changes on *E. faecium* growth by comparing the lag phase duration and doubling time of each isogenic RRDR mutant strain with that of the WT strain. Among the four mutant strains, the H486Y mutant showed a clear growth advantage, as evidenced by shorter lag and doubling times compared with the WT strain (Fig. 5A). The upregulation of nucleotide transport and metabolism-associated genes in this mutant might contribute to this phenotype. In contrast, both the S491L and Q473K mutants showed prolonged lag phases and doubling times, with the S491L mutant having the most severe growth defect of the four mutants tested. Interestingly, the G482V mutant displayed a mixed phenotype, with an extended lag phase but shorter doubling time relative to the WT strain (Fig. 5A). These growth differences could be due to the significant downregulation of carbohydrate transport and metabolism and translation-associated genes in these mutants (Fig. 4; File S1).

RRDR mutations have been known to influence susceptibility to antibiotics beyond those in the rifamycin class, in both *E. faecium* and other bacterial species (13, 14, 21, 32, 33). To assess these effects, we measured the minimum inhibitory concentrations (MICs) of various antibiotics against the WT and RRDR mutant strains. As expected, all four mutants displayed increased rifampin resistance compared to the WT strain (Fig. 5B). Additionally, all four mutants had decreased ampicillin MICs, ranging from 2-fold to 8-fold more susceptible. However, no differences in ceftriaxone susceptibility were noted, likely due to uniformly high MICs across all strains. Responses to meropenem and vancomycin were varied, with the G482V mutant displaying increased susceptibility to meropenem and the S491L mutant displaying increased resistance to vancomycin. Finally, the G482V and S491L mutant strains both had 2-fold higher MICs to daptomycin, whereas the Q473K mutant had a 2-fold lower daptomycin MIC compared with the WT strain. Together, these data suggest that RRDR mutations confer allele-specific effects on susceptibility to other antibiotics besides rifampin.

We also assessed differences in *E. faecium* fitness under antibiotic stress by determining the total area under the growth curve (AUC) in the presence of sub-MIC concentrations of the same antibiotics tested in Fig. 5B. Consistent with the slower growth rates of the Q473K, G482V, and S491L strains, these mutants also displayed lower growth under sub-MIC antibiotic pressure (Fig. 5C). The H486Y mutant, however, demonstrated better overall growth under meropenem stress, despite having the same MIC as the WT strain (Fig. 5C). Moreover, the H486Y mutant showed no significant reduction in growth under any antibiotic stress compared with the WT strain (Fig. 5C), perhaps due to the minimal transcriptional changes in this mutant (Fig. 3C). Finally, we investigated whether RRDR mutations contribute to isopropanol tolerance (34) and observed that the S491L mutant exhibited significantly higher tolerance to isopropanol compared with the WT strain after 15 but not 5 min of treatment (Fig. 5D; Fig. S4C). Together, these data suggest that the H486Y mutation and variation at residue 491 in the RRDR cause increased meropenem or isopropanol tolerance, respectively, either of which could facilitate the spread of *E. faecium* in clinical settings.

### Transcriptional changes in *E. faecium* carbohydrate metabolism genes are associated with differences in peptidoglycan abundance and muropeptide profiles

**Fig 6:**
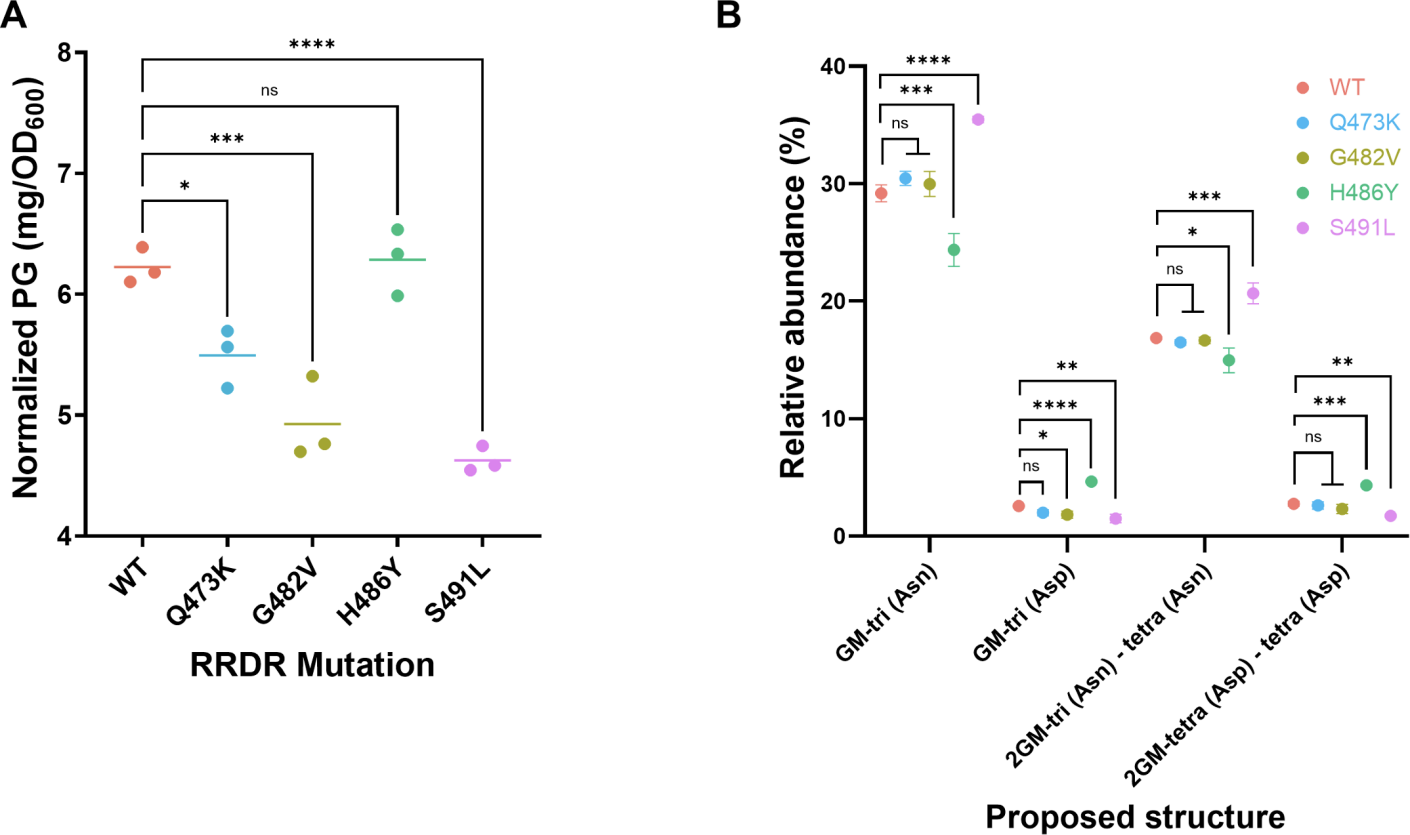
Changes in peptidoglycan abundance and composition due to RRDR mutation. (**A**) Normalized abundance of extracted peptidoglycan (PG) from wild type (WT) and mutant strains. (**B**) Relative abundance of muropeptides isolated from WT and mutant strains. GM, disaccharide (GlcNAc-MurNAc); 2 GM, disaccharide-disaccharide (GlcNAc-MurNAc-GlcNAc-MurNAc); GM-Tri, disaccharide tripeptide (L-Ala-D-iGln-L-Lys); and GM-Tetra, disaccharide tetrapeptide (L-Ala-D-iGln-L-Lys-D-Ala). Significance was calculated using one-way ANOVA with Tukey’s multiple comparison *post-hoc* test. *P ≤ 0.05; **P ≤ 0.01; ***P ≤ 0.001; ****P ≤ 0.0001; ns, not significant.

Given the changes we observed in mutant strain growth dynamics and expression of carbohydrate metabolism-associated genes, we wondered whether RRDR mutations also had altered peptidoglycan abundance and composition. The H486Y mutant, which displayed minimal transcriptional changes and growth defects, produced similar amounts of peptidoglycan as the WT strain (Fig. 6A). In contrast, the other three mutants, which all exhibited significant down-regulation of carbohydrate metabolism-associated genes, produced significantly less peptidoglycan compared with the WT strain (Fig. 6A). The S491L mutant, which exhibited the most dramatic transcriptional changes and growth defects, produced the least peptidoglycan of all mutants (Fig. 3D, 5A, and 6A). Together, these data suggest that RRDR mutations alter *E. faecium* peptidoglycan abundance in an allele-specific manner.

Further analysis of cell wall peptidoglycan fragments revealed significant differences in muropeptide composition between the WT and mutant *E. faecium* strains, particularly in amidation levels. The S491L mutant exhibited a significant increase in N-acetylglucosamine-muramyl tripeptide amidation compared with the WT strain (Fig. 6B and Fig. S5A; increased GM-tri (Asn) and decreased GM-tri (Asp)). This heightened amidation could result from altered intracellular glutamine levels due to the downregulation of *nodM* (LKHMGDEB_00431) and the upregulation of *gadB* (LKHMGDEB_00941), which utilize L-glutamine and L-glutamate as substrates, respectively (File S1) (35, 36). Increased amidation is often linked to greater peptidoglycan cross-linking, which can cause higher cell wall turnover and possibly a slower growth rate, as observed in the S491L mutant strain [Fig. 5A and 6B; increased 2GM-tri (Asn) – tetra (Asn)] (37, 38). Conversely, the muropeptide profile of the H486Y mutant showed significantly lower amidation levels. This might be associated with the upregulation of *glmS* (LKHMGDEB_00994), which also consumes L-glutamine as a substrate, thereby decreasing glutamine availability in the cell and overall amidation levels and potentially reducing the cost associated with cell wall synthesis and turnover (39) (Fig. 6B, File S1). This reduced metabolic cost might contribute to the faster growth rate observed for the H486Y mutant (Fig. 5A). We also compared the bacterial density (in CFU/mL) and total protein content of isogenic mutants to the WT strain to ensure that we harvested similar quantities of bacteria for peptidoglycan extraction. From cultures collected at the same OD_600_, we found no significant differences in total protein content or CFU/mL, suggesting that the samples contain similar biomass (Fig. S6A and B). Although further studies are needed to fully understand the connections between RRDR mutations, transcriptional changes, alterations in cell wall composition, and changes in growth rate and antibiotic susceptibility, these data nonetheless highlight the variable effects of different RRDR mutations on *E. faecium* physiology.

## DISCUSSION

To develop more effective anti-enterococcal therapeutics, a deeper understanding of how antimicrobial resistance mechanisms affect *E. faecium* physiology is critical. Here, we investigated the broader impacts of RRDR mutations beyond resistance to rifamycin antibiotics and their potential contribution to the global expansion of *E. faecium* as a hospital-adapted pathogen.

We found that *E. faecium* clinical isolates harboring RRDR mutations are prevalent and widely distributed both globally and locally. About 30% of global *E. faecium* genomes carried a mutation in the RRDR, with the most prevalent being S491F. The broad distribution of these mutations across various STs, countries, and genetic backgrounds, as well as the diversity of variants found, suggests that these mutations might confer significant advantages to the isolates harboring them. Further analyses of *E. faecium* clinical isolates from our local hospital showed that some RRDR mutations were associated with changes in susceptibility to daptomycin, a last-resort antibiotic used to treat vancomycin-resistant *E. faecium* infections. In contrast to a previous study (14), we found that the S491F mutation was inversely associated with daptomycin resistance in our local data set (Fig. 2C). This discrepancy might be influenced by different genetic backgrounds or by the fact that U.S. isolates predominantly encode *vanA*-type vancomycin resistance while Australian isolates encode *vanB*. (40) Additionally, daptomycin MIC testing was performed in our clinical microbiology laboratory on the Microscan platform, which might have overcalled resistance and impact the associations we identified.

RRDR mutations have been previously shown to affect *E. faecium* bacterial transcription by altering nucleotide binding and/or protein stability (14). In the isogenic mutants studied here, the G482V, H486Y, and S491L substitutions are predicted to introduce steric hindrance and disrupt hydrogen bonding in the RpoB active site, thereby affecting transcription efficiency. Meanwhile, the Q473K mutation replaces a neutral amino acid with a bulkier positively charged lysine, potentially causing electrostatic repulsion and altered RpoB activity. Transcriptomic and phenotypic analyses of these isogenic mutants revealed striking pleiotropic effects on *E. faecium* physiology. In general, RRDR mutations caused significant changes to *E. faecium* growth and gene expression profiles. Among the four mutants tested, the H486Y mutant stood out as the least disruptive, with a transcriptional profile most similar to WT. Moreover, this mutant exhibited slight increases in growth rate both without and with meropenem pressure compared with the WT strain, perhaps due to increased expression of nucleotide transport and metabolism-associated genes as well as decreased muropeptide cross-linking (37, 38). The combination of minimal collateral costs and improved growth rates likely contributes to the prevalence of this variant in clinical settings, with approximately 5% of global *E. faecium* isolates encoding this mutation. The Q473K, G482V, and S491L mutant strains, on the other hand, all displayed significant transcriptional disruptions, with the S491L mutant having the largest number of differentially expressed genes. All three mutants showed significant down-regulation of genes involved in carbohydrate metabolism, which likely led to the attenuated growth rates we observed in these strains. Muropeptide profiling revealed a significant increase in amidation levels in the S491L mutant strain, suggesting a higher metabolic cost during cell wall synthesis associated with increased muropeptide cross-linking that might further slow bacterial growth (37, 38). The RNA-seq data for the S491L mutant also pointed to increased intracellular glutamine levels as a possible driver of increased amidation. Notably, this mutant demonstrated increased tolerance to isopropanol, a likely selective advantage in nosocomial settings where isopropanol-based disinfectants are widely used. Although the S491L mutation is less prevalent than S491F, a prior study demonstrated that S491F produced phenotypic effects in *E. faecium* that are comparable to our findings with S491L, including a substantial fitness cost (14). Given these parallels, it is plausible that the S491F mutation might also confer increased isopropanol tolerance. Notably, the smaller number of genes with altered expression in the S491F mutant might explain the pervasiveness of this mutation rather than S491L in clinical settings, as well as its enrichment in emerging *E. faecium* lineages ST80 and ST117 (14–16).

In addition to their effects on growth dynamics and transcription, we also explored whether RRDR mutations influenced other phenotypes important for *E. faecium* pathogenicity, such as antibiotic susceptibility. All four RRDR mutant strains were more resistant to rifampin, but they were also all more susceptible to ampicillin, although we did not find significant changes in the mutants’ penicillin-binding protein five expression level or sequence (41). We were surprised to find that the S491L mutant had a higher vancomycin MIC compared with the WT strain. This mutant uniquely showed increased expression of a VanY-like D-alanyl-D-alanine carboxypeptidase, an enzyme involved in peptidoglycan remodeling and cell wall synthesis (File S1) (42, 43). Although primarily associated with cell wall synthesis, the function of this gene suggests a mechanism similar to VanY, whereby removal of the terminal D-alanine from the peptidoglycan pentapeptide prevents vancomycin binding, potentially providing a modest decrease in vancomycin susceptibility (43).

We also determined the impact of RRDR mutations on susceptibility to daptomycin, a frequently prescribed antibiotic for vancomycin-resistant *E. faecium* infections. Daptomycin resistance can be achieved through various mechanisms, including modifications to cell membrane composition and/or cell surface charge (44–47). Both the G482V and S491L mutants exhibited a 2-fold increase in daptomycin MIC; however, the mechanism by which this phenotype was likely achieved differed. Although the G482V mutant exhibited upregulated expression of *dltAB,* which has been shown to increase positive cell surface charge and thus decrease daptomycin binding (48–50), we did not observe increased positive cell surface charge in this mutant (File S1, Fig. S4D). This discrepancy could be due to additional changes in carbohydrate metabolism-associated genes in this mutant (Fig. 4). Regarding the decreased daptomycin susceptibility of the S491L mutant, we suspect that increased muropeptide amidation might contribute to reduced daptomycin binding to the cell membrane (44, 48, 49). Although the H486Y mutant exhibited upregulation of genes within the daptomycin resistance-associated LiaFSR system*,* these changes were not sufficient to increase daptomycin resistance, an observation consistent with a prior study (File S1) (46, 48, 51–53). We also investigated the *prdRAB* operon, which was recently implicated in RpoB-mediated daptomycin resistance in *E. faecium*. (14) However, no significant changes in *prdRAB* transcription were detected among any of the mutant strains we studied (File S1). This variability might reflect differences in genetic backgrounds or other differences between the vancomycin-susceptible strains used here and vancomycin-resistant *E. faecium* strains tested previously.

This study had several limitations. First, we were unable to fully recapitulate the most prevalent mutations identified among global *E. faecium* isolates. Given the allele-specific changes we observed, it is unknown whether a different mutation at the same amino acid residue would cause similar effects on *E. faecium* physiology. Second, although each of the RRDR mutations we studied conferred distinct selective advantages *in vitro*, their role in *E. faecium* growth and survival during infection remains to be investigated. Finally, it is unclear how the presence of vancomycin resistance plasmids might influence the effects of RRDR mutations, especially since some of our findings were discrepant with those of a recent study (14). Nonetheless, our findings are in agreement with prior reports showing that alterations in the RRDR lead to collateral impacts on *E. faecium* growth, gene expression, and antibiotic susceptibility (13, 14, 18, 20, 28, 30, 33, 54).

Overall, this study illustrates how different RRDR mutations conferring rifampin resistance have unique and allele-specific effects on *E. faecium* physiology, highlighting the complex interplay between RRDR mutations and transcriptional and phenotypic changes. Understanding how RRDR mutations emerge and spread can help inform novel targets for therapeutic strategies to mitigate *E. faecium* persistence and pathogenicity in hospital settings.

## MATERIALS AND METHODS

### Global *E. faecium* isolate analysis

All *E. faecium* genomes deposited in the NCBI between 2000 and 2023 were downloaded on 26 March 2024. Genomes were filtered as described in Fig. S1. Briefly, only genomes with “geo_location,” “host,”, and “isolation_source” entries were included. Samples were de-duplicated based on BioSample accession, and only those for which “host” included *Homo sapiens, Homo sapiens sapiens,* or human were retained for further analysis. To avoid contamination or incomplete assemblies, genomes with a size of >2.6 Mbp and <3.3 Mbp (QUAST v5.2.0 (55)), and which were not isolated from the environment, were used for the final global data set (total = 14,384 isolates). Multi-locus sequence types (STs) were identified using the PubMLST database (56). RRDR variants were determined using Geneious v2024.0.3 by aligning sequences to a representative WT RRDR sequence (467-GSSQLSQFMDQTNPLGELTHKRRLSAL-493).

### Local *E. faecium* isolate analysis

In total, 710 vancomycin-resistant *E. faecium* isolates were collected between 2017 and 2022 as part of a retrospective observational study at the University of Pittsburgh Medical Center called the Enhanced Detection System for Healthcare-Associated Transmission (EDS-HAT) (17). Antibiotic exposure data were assessed for the 90 days prior to isolate collection. Daptomycin susceptibilities were determined through automated testing on the MicroScan platform and were interpreted according to the 2025 Clinical and Laboratory Standards Institute M100 guidelines (57). Whole-genome sequencing was performed on the NextSeq500 platform (Illumina, San Diego, CA). The resulting reads were assembled using SPAdes v3.15.5 and annotated with Prokka v1.14.5 (58, 59). Clinical isolate genomes were analyzed using the same pipeline as above for global *E. faecium* isolates.

### Bacterial growth conditions and selection of RRDR mutant strains

All *E. faecium* strains were grown in BHI (Brain Heart Infusion, Difco) broth aerobically at 37°C with agitation at 170 rpm. Overnight cultures were inoculated into 3 mL media and grown for 18 h. For *in vitro* rifampicin resistance selection, a previously described ST203 *E. faecium* strain called DVT705 was used as the parent wild-type (WT) strain, with antibiotic susceptibilities summarized in File S1 (16). To select isogenic strains with RRDR mutations, an overnight culture of DVT705 was plated onto several BHI agar plates containing 50 µg/mL rifampin. At least three different colonies were picked from each plate, and the RRDR region was Sanger-sequenced. Four isolates with unique mutations were identified and further characterized. To evaluate the isogenic mutant strains with whole genome sequencing, genomic DNA was extracted using a DNeasy Blood and Tissue Kit (Qiagen, Germantown, MD) according to the manufacturer’s protocol with additional incubation with 10 µL of 2500 U/mL mutanolysin and 50 µL 50 mg/mL lysozyme at 37°C for 1 h. Next-generation sequencing libraries were prepared (2 × 150 bp, paired-end reads) and sequenced on the Illumina platform at SeqCenter (Pittsburgh, PA). The resulting reads were assembled using SPAdes v3.15.5 and annotated with Prokka v1.14.5 (58, 59). Variants in each isogenic mutant were confirmed using breseq v0.37.1 (60).

### *E. faecium* growth dynamics and antimicrobial susceptibility testing

Changes in growth rates and antibiotic minimum inhibitory concentrations (MICs) of WT and isogenic mutant strains were determined as follows. Briefly, overnight cultures were normalized to an OD_600_ of 0.05, and 5 µL of the diluted culture was used to inoculate 200 µL fresh BHI media in 96-well microplates (1:40 dilution). OD_600_ readings were taken every 20 min, interspersed with 5 min of orbital shaking at 37°C for 24 h using a Synergy H1 microplate reader (Biotek, Winooski, VT). For lag time calculations, the start of log-phase growth was set to a threshold of OD_600_ = 0.1. Antibiotic MICs were determined using the broth microdilution method (57). Daptomycin MICs were determined with the addition of 50 µg/mL CaCl_2_ to the media. To assess growth in the presence of sub-MIC antibiotic concentrations, the following were tested: ampicillin at 128 µg/mL, ceftriaxone at 8,192 µg/mL, meropenem at 128 µg/mL, vancomycin at 1 µg/mL, and daptomycin at 8 µg/mL. The results are presented as heatmaps showing log_2_ fold-changes relative to the WT strain. Growth curves without antibiotic pressure are shown in Fig. S6C.

Isopropanol tolerance was determined as previously described (34). Briefly, overnight cultures were diluted to an OD_600_ of 1.66 in fresh BHI. Either 1 mL of phosphate-buffered saline (PBS; positive control) or isopropanol at 20% final concentration was added to 300 µL of the diluted cultures. Samples were vortexed briefly and incubated at room temperature for five or 15 min without shaking. Just prior to dilution, samples were vortexed again briefly. Serial dilutions (10^-1^ to 10^7^) were prepared in PBS containing 7.5% Tween 80 and were spotted onto BHI plates to enumerate the colony-forming units per mL (CFU/mL). Plates were incubated overnight at 37°C, and CFU/mL were compared between PBS-treated and isopropanol-treated samples. Percent survival was calculated as the ratio of CFU/mL of isopropanol-treated samples to CFU/mL of PBS-treated samples. All experiments were conducted in biological triplicate.

### RNA sequencing and analysis

RNA was extracted from WT and isogenic mutant strains using a RNeasy Mini Kit (Qiagen) according to the manufacturer’s protocol with minor modifications. Briefly, overnight cultures were re-inoculated to 5 mL fresh BHI at a 1:100 dilution factor and grown until mid-log phase (OD_600_ 0.3–0.4); 100 µL of the mid-log phase culture was serially diluted, plated on BHI plates, and incubated at 37°C overnight to enumerate colony-forming units per mL (CFU/mL). Then, 2× vol/vol of RNAprotect (Qiagen) was added to each leftover culture, and the cells were pelleted. The pellet was incubated with 10 µL of 2,500 U/mL mutanolysin and 50 µL 50 mg/mL lysozyme at 37°C for 30 min prior to RNA extraction. Total RNA was treated with DNase, and 2 × 150 bp paired-end libraries were generated and sequenced on an Illumina NovaSeq X Plus (SeqCenter, Pittsburgh, PA). Four replicate samples were collected for each strain. Sequencing reads were processed using Trim Galore v0.6.10 (61). Reads passed quality control if the Phred score was >33 and the sequence length was >142 bp. Trimmed reads were then mapped to the annotated WT genome using STAR v2.7.11b (62). Reads mapping to coding sequences were counted using featureCounts v2.0.6 against the annotated WT sequence (63). Differential gene expression analysis was conducted using the DESeq2 package in RStudio (64). For reliability, only genes with read counts > 10 were included in the analysis. Genes were considered significantly differentially expressed if they had an adjusted *P*-value < 0.05 (Benjamini-Hochberg procedure applied for false discovery) and log_2_ fold-change values <−1 or >1. Data were normalized with the varianceStabilizingTransformation() function for principal component analysis (PCA). Volcano plots and Venn diagrams were constructed using the EnhancedVolcano and VennDiagram packages in RStudio, respectively.

The Clusters of Orthologous Groups (COG) database was used to assign functions to differentially expressed genes using EggNOG-mapper (25, 26, 65). Approximately 84% (2388/2854) of annotated genes in the WT strain were assigned to a COG category. For distribution analysis, genes assigned to multiple COG categories were counted once in each category to yield a total of 2,506 categorized genes. DEGs without a specified COG category were excluded from analysis.

### Peptidoglycan LC-MS analysis

Peptidoglycan was extracted from the sacculi of WT and isogenic mutant strains. Briefly, overnight cultures were diluted 1:100 and grown to OD_600_ 0.6–0.8, and then, the cultures were treated with 1M HCl to remove teichoic acid and digested with 10 KU/mL mutanolysin, as previously described to obtain soluble peptidoglycan (66). For LC-MS analysis, the peptidoglycan of each strain was further analyzed as previously described (67). Briefly, mutanolysin-digested soluble peptidoglycan was treated with sodium borohydride in 0.25 M boric acid at pH 9 for 1 h at room temperature. Orthophosphoric acid was then used to quench the reaction, and the pH was adjusted to 2–3. After centrifugation at 20,000 × *g* for 10 min, peptidoglycan fragments were separated and analyzed on a 1290 Infinity II LC/MSD system (Agilent Technologies) using a Poroshell 120 EC-C18 column. The mobile phase consisted of 0.1% formic acid in water, and the eluent was 0.1% formic acid in acetonitrile. Samples were first run against the mobile phase at a flow rate of 0.5 mL/ min, then against 2% (0–5 min) and 2%–10% (5–65 min) eluent. Absorbance of the eluting peaks was measured at 205 nm and detected with MSD API-ESI Scan mode (*m*/*z* = 200–2,500). The area under the curve of each individual peak from the chromatogram was calculated, and the relative abundance of each muropeptide was calculated as the percentage of each individual peak relative to all assigned peaks. The experiment was performed on three biological replicates.

### Bradford protein assay

The Bradford assay was performed to determine the total protein content of the WT and isogenic mutants. Overnight cultures were re-inoculated at 1:100 dilution and grown until OD_600_ 0.6–0.8 prior to harvesting. All cultures were then normalized to OD_600_ = 0.6, washed with TE buffer, and incubated with 10 µL of 2,500 U/mL mutanolysin and 50 µL 50 mg/mL lysozyme at 37°C for 1 h. The supernatant was then used for Bradford protein assay (Bio-Rad) according to the manufacturer’s protocol. This assay was conducted in biological and technical triplicates.

### Cytochrome C binding assay

Cytochrome C binding was performed on WT and isogenic mutant strains as previously described with minor modifications (68). Briefly, overnight cultures were washed three times with 1 mL of 20 mM 3-(N-morpholino) propanesulfonic acid sodium salt buffer (MOPS, pH = 7.0) and normalized to OD_600_ = 0.2. The normalized suspension was then incubated with an equal volume of 1 mg/mL cytochrome C (Sigma Aldrich). After 30 min incubation at room temperature, samples were centrifuged for 5 min at 13,000 rpm. The absorbance of the supernatant was measured at 410 nm in a Synergy H1 microplate reader (Biotek, Winooski, VT), and the results were averaged across technical triplicates. This experiment was conducted in biological triplicate.

### Statistical analysis

Significance for COG enrichment, associations between RRDR mutations with prior rifamycin class exposure, and daptomycin susceptibility were assessed using Fisher’s exact test, with Bonferroni correction when appropriate. Significance for growth rate, isopropanol tolerance, peptidoglycan analysis, and cytochrome C binding assays was assessed using one-way ANOVA, with Tukey’s multiple comparison post-hoc test when appropriate. All calculations were conducted in GraphPad Prism (v.10.0).

## Data Availability

Genomes from the global collection are listed in File S1. Genomes from the local collection are deposited in NCBI under BioProject PRJNA475751. RNA-sequencing data are deposited in the Gene Expression Omnibus (GEO) at NCBI under accession GSE302807.

## SUPPLEMENTAL MATERIAL

Supplemental Figures msphere.00506-25-s0001.pdf Figures S1-S6.

Supplemental Data Set msphere.00506-25-s0002.xlsx File S1.

ASM does not own the copyrights to Supplemental Material that may be linked to, or accessed through, an article. The authors have granted ASM a non-exclusive, world-wide license to publish the Supplemental Material files. Please contact the corresponding author directly for reuse.
