# Supplementary material for: Allele-specific effects of mutations in the rifampin resistance-determining region (RRDR) of RpoB on physiology and antibiotic resistance in Enterococcus faecium
Source: mSphere. 2025 Dec 4;10(12):e00506-25. doi: 10.1128/msphere.00506-25 (PMC12724266; doi:10.1128/msphere.00506-25)
Supplement: Supplemental Figures — Figures S1-S6. [file msphere.00506-25-s0001.pdf]

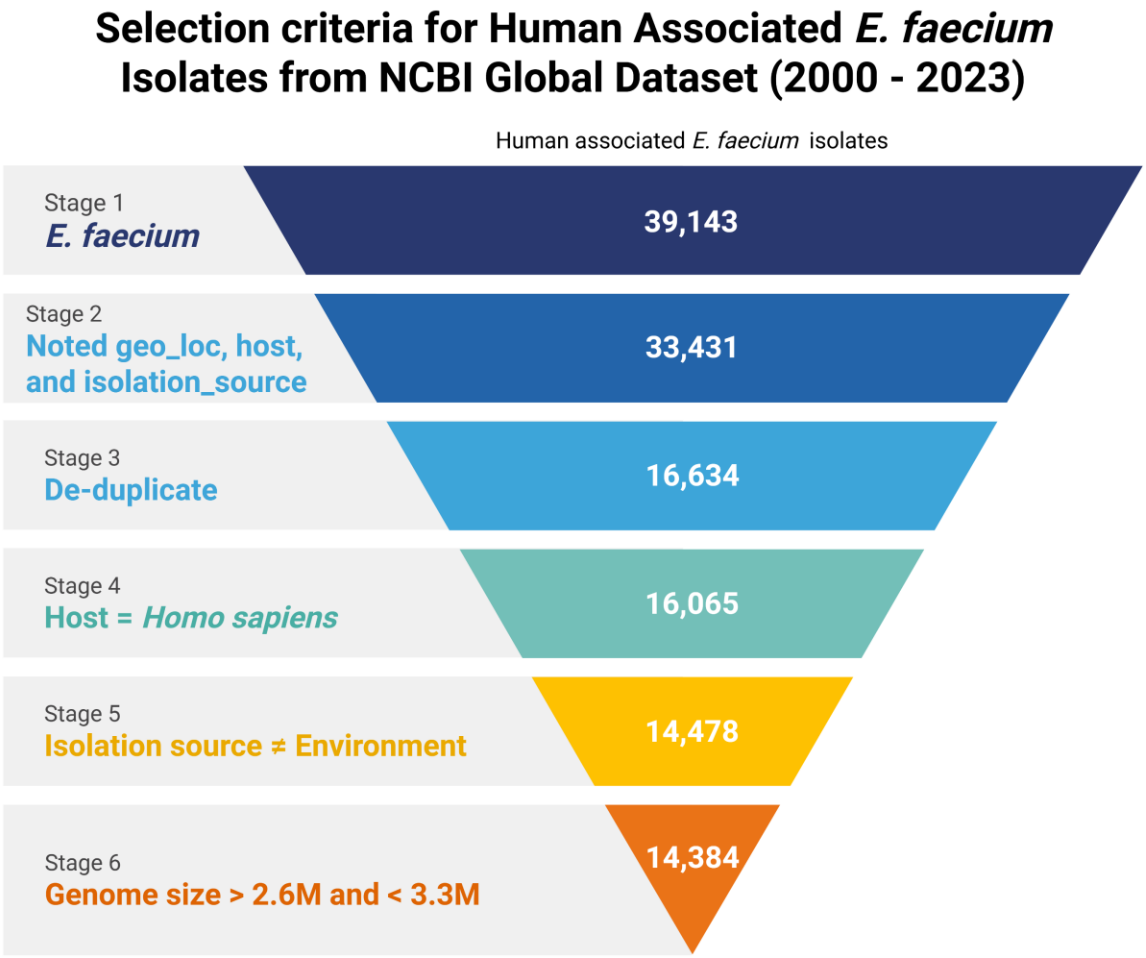

Figure S1. Selection criteria for human-associated *E. faecium* isolate genomes deposited in NCBI collected between 2000 and 2023. A total of 39,143 genomes deposited as *Enterococcus faecium* between 2000 and 2023 with noted geo location, host, and isolation source were downloaded on March 26th, 2024. After de-duplication, only assemblies with "host" of "*Homo sapiens*", "*Homo sapiens sapiens*", "human", or "human being" were retained. We further selected for genomes that were not isolated from the environment and have genome sizes between 2.6 Mbp and 3.3 Mbp. Final number of assemblies included was 14,384.

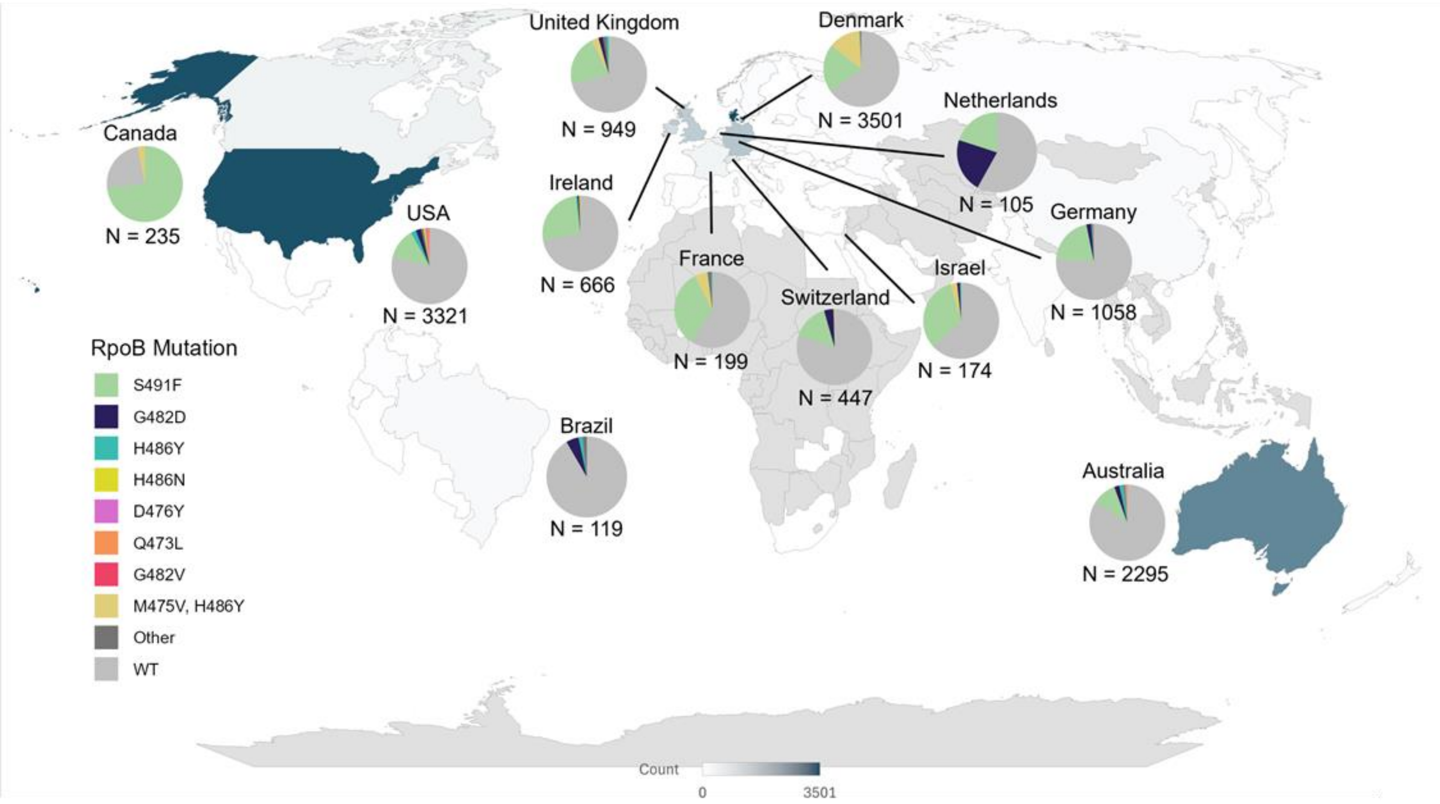

Figure S2. Geographical distribution of vancomycin-resistant *E. faecium* genomes with RRDR mutations deposited in NCBI. Number of genomes from each country is shown from lowest in light grey to highest (blue). Pie charts show distribution of mutations among isolates from countries with >100 genomes.

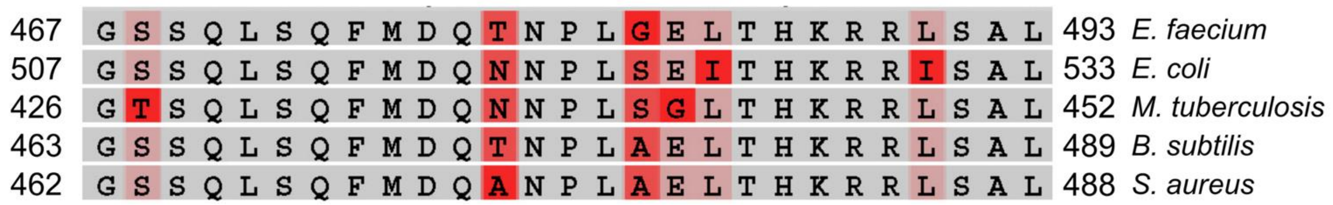

Figure S3. Alignment of RRDR region across bacterial species. Alignment was conducted using Constraint-based Multiple Alignment Tool (COBALT). RRDR protein sequence from *E. faecium* is from the wild type strain used in this study. Sequences of the other species were obtained from NCBI: *E. coli* (AJF45152.1), *M. tuberculosis* (VCU48907.1), *B. subtilis* (COO12072.1), *S. aureus* (WRN42392.1). Red shading depicts residues with differences in sequence between species.

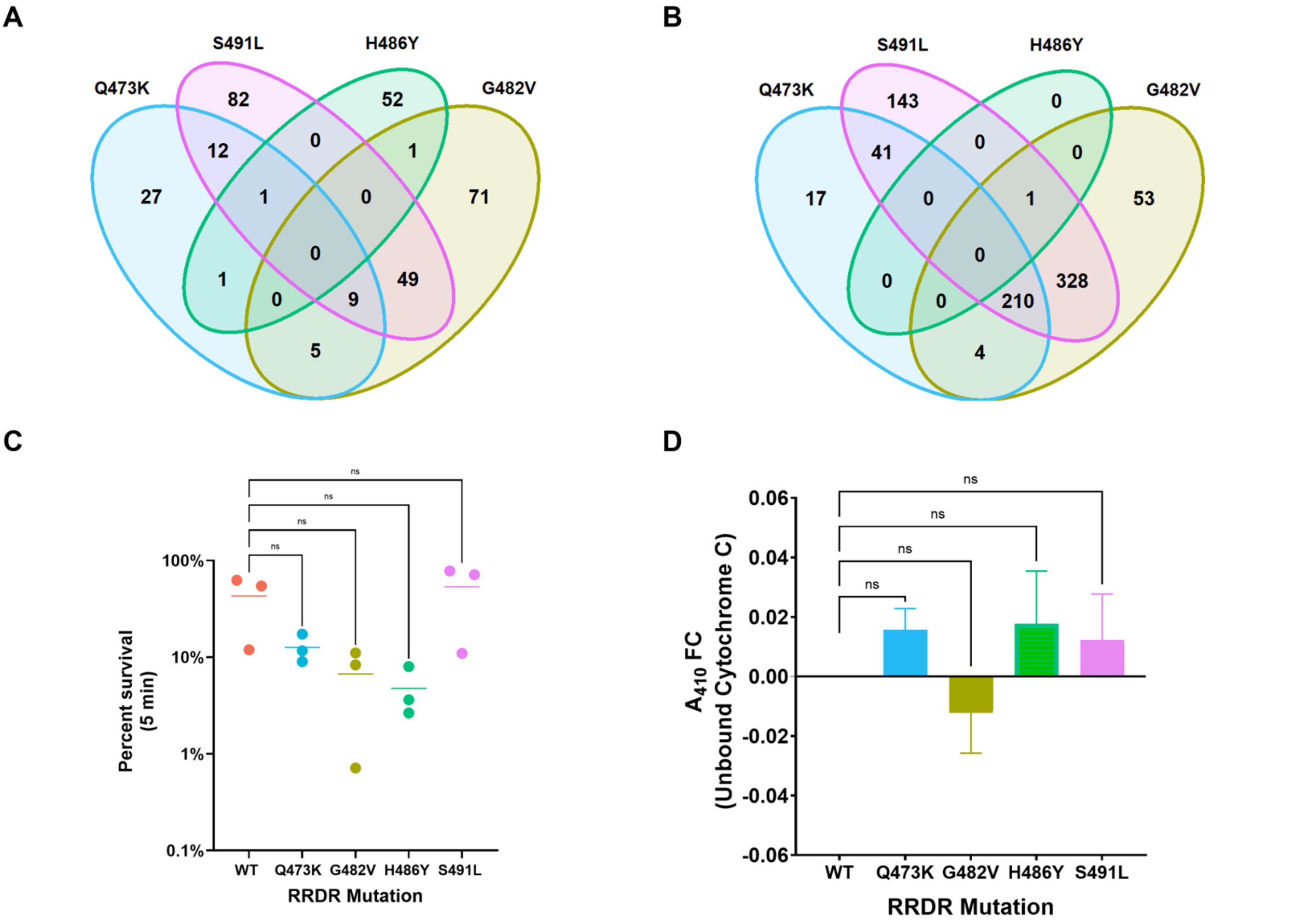

Figure S4. (A) Overlap in differentially expressed genes with upregulated expression between RRDR mutant strains. (B) Overlap in differentially expressed genes with downregulated expression between RRDR mutant strains. (C). Percent survival of *E. faecium* strains after incubation with 20% isopropanol for 5 min. (D) Cell surface charge determined through binding of cytochrome C. More unbound cytochrome C denotes a more positively charged cell surface. Experiments were conducted in triplicate. Significance was assessed using one-way ANOVA. ns, not significant.

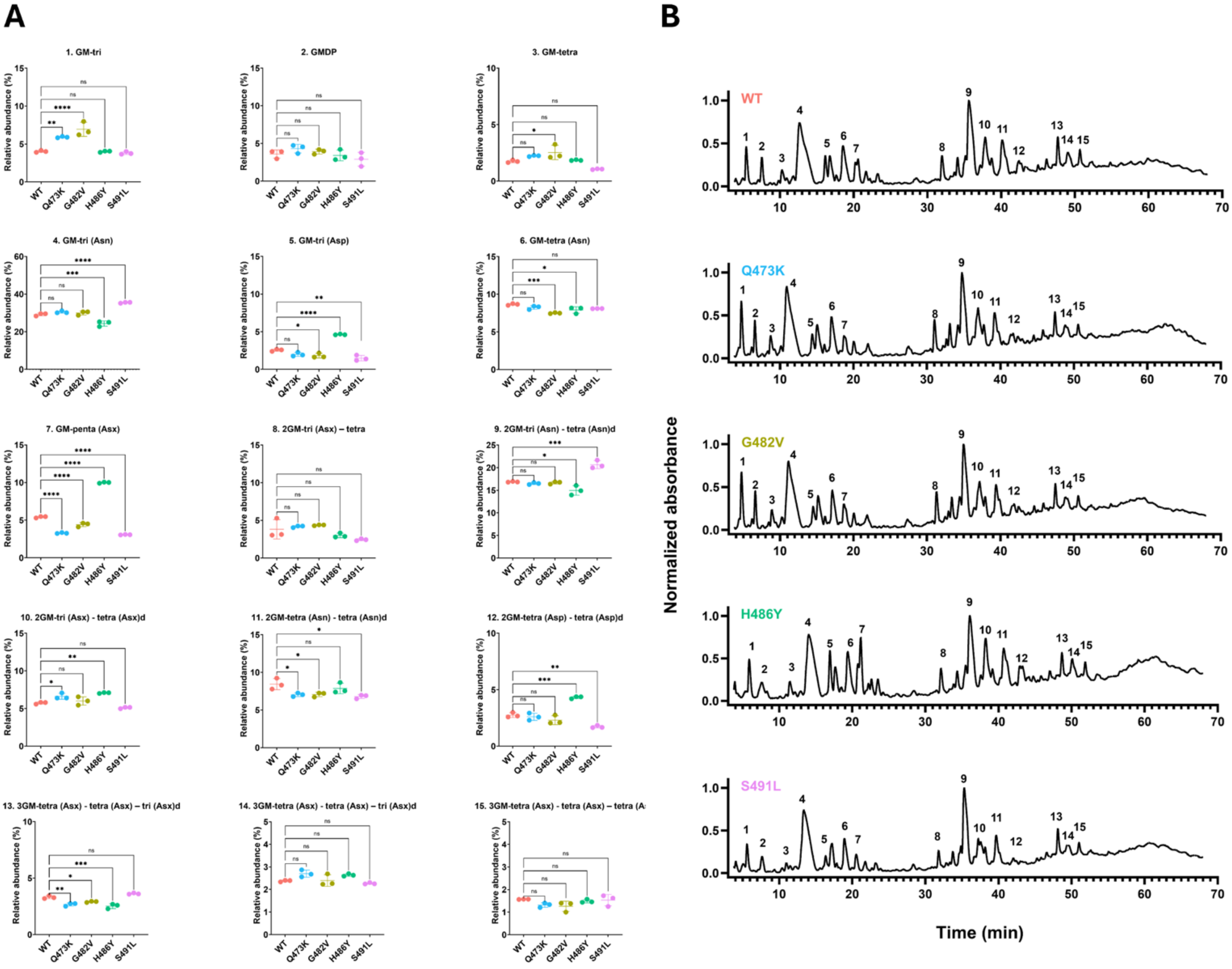

Figure S5. (A) Relative muropeptide abundance of wild type (WT) and RRDR mutant strains. (B) Representative LC-MS chromatograms of mutanolysin-digested peptidoglycan isolated from sacculi of WT and RRDR mutant strains. GM, disaccharide (GlcNAc-MurNAc); 2GM, disaccharide-disaccharide (GlcNAc-MurNAc-GlcNAc-MurNAc); 3GM, disaccharide-disaccharide-disaccharide (GlcNAc-MurNAc-GlcNAc-MurNAc-GlcNAc-MurNAc); GM-Tri, disaccharide tripeptide (L-Ala-D-iGln-L-Lys); GM-Tetra, disaccharide tetrapeptide (L-Ala-D-iGln-L-Lys-D-Ala); GM-Penta, disaccharide pentapeptide (L-Ala-D-iGln-L-Lys-D-Ala -D-Ala). Significance was calculated using one-way ANOVA with Tukey's multiple comparison post-hoc test. *p≤0.05; **p≤0.01; ***p≤0.001; ****p≤0.0001; ns, not significant.

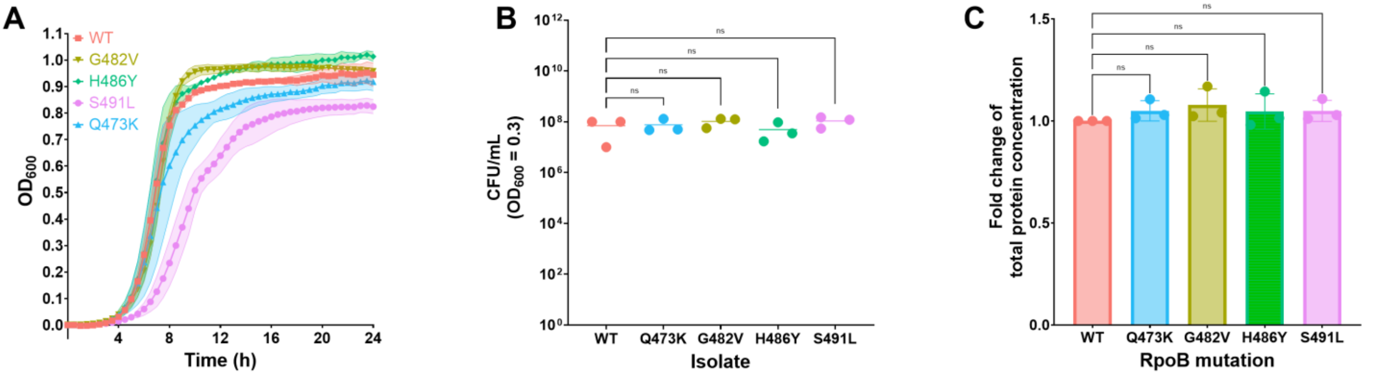

Figure S6. (A) Growth curves of the WT and isogenic mutants without antibiotic pressure. (B) Colony forming unit (CFU) determination at OD_{600} = 0.3. (C). Bradford assay to calculate total protein concentration of the WT and isogenic mutants. Significance was assessed using one-way ANOVA. ns, not significant.
